# The Gill-Associated Bacterial Community Is More Affected by Exogenous *Chlorella pyrenoidosa* Addition than the Bacterial Communities of Water and Fish Gut in GIFT Tilapia (*Oreochromis niloticus*) Aquaculture System

**DOI:** 10.3390/biology12091209

**Published:** 2023-09-05

**Authors:** Shunlong Meng, Huimin Xu, Lu Qin, Xi Chen, Liping Qiu, Dandan Li, Chao Song, Limin Fan, Gengdong Hu, Pao Xu

**Affiliations:** 1 Freshwater Fisheries Research Center, Chinese Academy of Fishery Sciences, Scientific Observing and Experimental Station of Fishery Resources and Environment in the Lower Reaches of the Yangtze River, Wuxi 214081, China; mengsl@ffrc.cn (S.M.); xuhuimin@ffrc.cn (H.X.); chenx@ffrc.cn (X.C.); qiulp@ffrc.cn (L.Q.); lidd@ffrc.cn (D.L.); songc@ffrc.cn (C.S.); fanlm@ffrc.cn (L.F.); hugd@ffrc.cn (G.H.); 2 Wuxi Fishery College, Nanjing Agricultural University, Wuxi 214081, China; qinlu121212@163.com

**Keywords:** tilapia aquaculture, *Chlorella pyrenoidosa* addition, different microhabitats, bacterial communities, community characteristics

## Abstract

**Simple Summary:**

Microorganisms are important components of biological communities in the aquaculture ecosystems, which play fundamental roles in regulating the water quality and maintaining the health of cultured animals. Microalgae addition as a common aquaculture practice has been reported to have positive effects on both the aquaculture environment and organisms. However, the ecological effects of microalgae addition on the aquaculture ecosystems from the perspective of microbial community ecology are still unclear. In the present study, we analyzed the characteristics of microbial communities in various microhabitats in tilapia aquaculture systems with different microalgae additions. It was found that microalgae addition exhibited greater impacts on the diversity, compositions, and co-occurrence patterns of the gill-associated bacterial communities than those of water and fish gut. The mucosal surface of the fish gill represented an important barrier essential for fish health. Microalgae addition altered the composition of gill-associated bacterial communities, which could be important for mucosal immunity and thereby influence fish health. These findings provided a new insight into the mechanisms underlying the impacts of microalgae addition on aquaculture.

**Abstract:**

Microalgae has been widely used in aquaculture to improve both the water environment and fish growth; however, the current understanding of the effects of microalgae addition on the key players involved in regulating the water environment and fish health, such as microorganisms, remains limited. Here, a 50-day mesocosm experiment was set up to simulate the culture of Genetic Improvement of Farmed Tilapia (GIFT, *Oreochromis niloticus*) with an average weight of 14.18 ± 0.93 g and an average length of 82.77 ± 2.80 mm. Different amounts of *Chlorella pyrenoidosa* were added into these artificial systems to investigate dynamics of bacterial communities in aquaculture water, fish gill, and gut using amplicon-based high-throughput sequencing technology. Our results showed that *Chlorella pyrenoidosa* addition increased diversity and network complexity of gill-associated bacterial communities rather than those of the water and gut. Furthermore, more biomarkers in the gill-associated bacterial communities were detected in response to *Chlorella pyrenoidosa* addition than the water and fish gut samples. These findings highlighted the high sensitivity of gill-associated bacterial communities in response to the *Chlorella pyrenoidosa* addition, implying *Chlorella pyrenoidosa* addition could play important roles in regulating the fish mucosal immunity by altering the gill-associated microbiota.

## 1. Introduction

As primary producers in aquatic ecosystems, microalgae play an important role in biogeochemical processes in aquaculture systems. Microalgae have been employed in the treatment of aquaculture wastewater, as well as in the regulation of in situ aquaculture environments [1,2]. During the aquaculture period, microalgae can also be used as bait to supply biological energy for the cultured animals [3]. It has been reported that microalgae can effectively improve the immune function and growth of the cultured animals, thereby increasing the efficiency of aquaculture [4,5]. Among them, *Chlorella pyrenoidosa*, belonging to the genus *Chlorella*, phylum Chlorophyta, is widely used in feed addition in aquaculture because of it contains a variety of unsaturated fatty acids, proteins, carotenoids, biological polysaccharides, minerals, and other metabolites [6]. Therefore, the exogenous microalgae addition can achieve the dual effects of regulating water quality and promoting the growth of the cultured animals, thereby providing an effective way to promote sustainable development of freshwater aquaculture. Although the positive effects of the exogenous microalgae addition on improving both water quality and growth of the cultured animals have been addressed [7,8], the impacts of the exogenous microalgae addition on the key players in the aquaculture systems, such as microbial communities, are rarely considered [9].

Microorganisms play an important role in aquaculture processes, as they are essential for biogeochemical processes as well as the health of cultured animals. Aquaculture systems are characterized as environments with enormous biomass, high turnover rates, and environmental complexity, which provide an extreme genetic diversity inhabiting various microhabitats [10]. In the simplest aquaculture system, the microorganisms could be either located in the water environment or associated with the aquatic animals (i.e., hosts). In the water environment, the interaction between the algae and bacteria are quite complex [11], which could be based on the provision of resources and can be either reciprocal or exploitative [12]. For their symbiotic cooperation relationship, it has been shown that algae can provide polysaccharide substances for bacterial growth; in turn, bacteria can produce vitamins for algal growth [11]. Algae and bacteria also have obvious competition mechanisms for inorganic nutrients such as nitrogen and phosphorus. The bacterial community diversity and composition can be affected by even minor changes in algal populations, which may affect the mineralization of complex organic matter in water [13]. Thus, the exogenous microalgae addition could be beneficial for the temporary removal of inorganic nitrogen and phosphorus, as the microalgae can utilize these inorganic nutrients. Then, the aquatic bacterial communities structured by the exogenous microalgae addition would have long-term effects on the water quality of the aquaculture environment. Host-associated microbial communities are also important components of aquaculture microbiota, which can often be seen as an extension of the host genome by providing functions critical for the host survival [14]. Fish are the most common animals in aquaculture systems worldwide. The microbes present in a fish’s gill determine how the fish recognize and react to pathogens through their mucosal surfaces [15]. Furthermore, fish gut microbes play a vital role in the nervous system development, behavior, immunity, food digestion, and metabolism of the host [16]. The assessment of the fish gill- and gut-associated microbiomes will provide information about fish health and the identification of microorganisms that could be significant bio-indicators. In turn, fish gill- and gut-associated microbiomes can be influenced by the water environment and the microbial communities derived from the water environment [17,18]. On one hand, the addition of exogenous microalgae could affect the fish gill- and gut-associated microbiome indirectly by regulating the water quality and shaping the bacterioplankton communities. On the other hand, the addition of exogenous microalgae could also exhibit impacts on the gut microbiome by changing the diet of fish. To date, however, we still lack an understanding of the impacts of exogenous microalgae addition on the bacterial communities inhabiting different microhabitats (i.e., water, gill, and gut) of the aquaculture systems.

The rapid development of high-throughput sequencing technology has provided researchers with a deeper understanding of the diversity, composition, and function of microbial communities in the environment. In recent years, there has been increasing research focusing on the diversity and composition of microbial communities in aquaculture environments, including bacterioplankton [19], fish-gill-associated microbiomes [20], and fish gut microbiomes [21]. However, microbes do not exist independently in the environment; the symbiotic or competitive relationships between species are not only crucial for maintaining community diversity, but also play an important role in the functioning of aquaculture systems. Currently, network analysis provides us with a mathematical tool to explore the potential interactions between species in microbial communities [22], which has been widely used in the field of aquaculture microbiomes [23,24]. The detected positive or negative correlations, based on data of taxonomic abundance, can provide insights into co-occurrence/exclusion relationships between taxonomic groups [25]. However, whether aquaculture practices of the exogenous microalgae addition affect the co-occurrence patterns of bacterial communities in different microhabitats (i.e., water, fish gill, and gut) remains unknown. Investigating the network relationships of bacterial communities in different microhabitats can provide new perspectives for understanding the impact of exogenous microalgae addition on aquaculture systems, which is of great significance.

In this study, Genetic Improvement of Farmed Tilapia (GIFT, *Oreochromis niloticus*) was used as the cultured species, and a 50-day mesocosm experiment was conducted to investigate the microbial community dynamics of water, fish gill, and gut using amplicon-based high-throughput sequencing technology under scenarios with different microalgae (*Chlorella pyrenoidosa*) additions. The following questions will be addressed: (1) How does the *Chlorella pyrenoidosa* addition affect the diversity, composition, and function of bacterial communities in water, fish gill, and gut? (2) How does the *Chlorella pyrenoidosa* addition influence the bacterial community networks in water, fish gill, and gut? The obtained results can provide insights for the long-term ecological effects of using microalgae in aquaculture processes.

## 2. Materials and Methods

### 2.1. Experimental Design

The mesocosm experimental systems were set up using 1000 L circular polyethylene barrels to simulate the process of tilapia aquaculture from July to September, 2020. A total of 800 L of tap water was added to each experimental system, and air pumps were used to aerate the water for 7 days before the culture experiment. The experimental fish used were Genetic Improvement of Farmed Tilapia (GIFT, *Oreochromis niloticus*), provided by the tilapia breeding test base of the Freshwater Fisheries Research Center of the Chinese Academy of Fishery Sciences. Healthy tilapia with uniform size, strong physique, no illness or injury, and normal swimming were selected for the experiment. The microalgae used was *Chlorella pyrenoidosa*, provided by the Freshwater Algae Culture Collection of the Chinese Academy of Sciences (FACH). Then, *Chlorella pyrenoidosa* was cultivated with BG11 liquid medium to expand the cultures in the laboratory, which resulted in a microalgal solution with a concentration of 1 × 10^9^ cells/L. Three experimental treatments were set up with 50 mL, 500 mL, and 5000 mL microalgal solutions added into the experimental systems every 10 days. These are referred to as the low-concentration group (LC), the medium-concentration group (MC), and the high-concentration group (HC). The compositions and concentrations of the *Chlorella pyrenoidosa* additive solutions were determined under a microscope. A control treatment was also set up without the addition of microalgal solution (NC). Each treatment had three replicates, with 30 fish in each. Fish were fed twice a day, at 9:00 a.m. and 4:00 p.m., with a commercial diet (crude protein ≥ 40%, crude fat ≥ 12%, coarse fiber ≤ 6%, crude ash ≤ 18%; TONGWEI CO., LTD, Chengdu, China), with a feeding amount equivalent to ~3% of the fish body weight in the culture barrel (estimated based on the weight of six fish randomly selected from each group during each sampling). An air pump was used for aeration throughout the day. To minimize the impact on aquatic microorganisms during the culture process, water was not exchanged throughout the experiment. However, due to evaporation caused by weather and other factors, water that was lost was refilled to the initial volume after each sampling.

### 2.2. Sampling Work and Measurements of Water Quality

Water and fish samples were collected on day 20, 40, and 50 of the culture experiment for the determination of water quality and microbial community compositions. A water sample of 500 mL was filtered through a 0.2 μm pore, 47 mm diameter polycarbonate membrane (Millipore, Billerica, MA, USA), and the membrane was then shredded with sterile scissors before being placed into sterilized tubes. Two fish samples were collected from each replicate system for each sampling timepoint. Firstly, fish were immediately euthanized by use of a lethal dose of tricaine mesylate (MS222). The gill was removed from the gill chamber with sterile scissors, then placed into labelled sterilized polypropylene centrifuge tubes filled with liquid nitrogen. The intestines were aseptically removed from their abdominal cavity; then, the luminal content was retrieved from the intestines and placed into labelled sterilized polypropylene centrifuge tubes. All of the samples were stored at −80 °C before DNA extraction.

Unfiltered water samples were used to measure the total nitrogen (TN), total phosphorus (TP), and chemical oxygen demand (determined by potassium permanganate titration, COD_Mn_). Furthermore, the filtrate was used to determine the ammonia nitrogen (NH_4_^+^-N), nitrate nitrogen (NO_3_^−^-N), and nitrite nitrogen (NO_2_^−^-N). The measurements were processed according to according to the national standard methods.

### 2.3. DNA Extraction, PCR, and Sequencing

DNA was extracted using the Omega Biotek EZNA stool DNA Kit (Georgia, USA). The final DNA concentration was evaluated using a NanoDrop 2000 UV–vis spectrophotometer (Thermo Scientific, Wilmington, NC, USA), and DNA quality was assessed by 1% agarose gel electrophoresis. The V3-V4 region of the 16S ribosomal RNA gene of the bacteria was amplified using primers 338F (5′-ACTCCTACGGGAGGCAGCAG-3′) and 806R (5′-GGACTACHVGGGTWTCTAAT-3′) in a polymerase chain reaction (PCR) [26]. The PCR reaction mixture and thermal cycling conditions were consistent with the existing procedures [19]. For each sample, PCR was performed three times. The Illumina paired-end library was constructed, and the amplicons were then sequenced using the Illumina Miseq PE 300 platform (San Diego, CA, USA). The raw reads were submitted to the NCBI Sequence Read Archive (SRA) database (Bioproject ID: PRJNA997058). The bioinformatics of these paired-end raw sequences are provided in the supplementary materials.

### 2.4. Statistical Analysis

The observed OTUs, phylogenetic diversity, and Pielou’s evenness were calculated as alpha diversity of bacterial communities using packages ‘vegan’ and ‘picante’ in R [27,28]. The impacts of microhabitats (i.e., water, fish gill, and gut), treatments (different *Chlorella pyrenoidosa* additions), and sampling time on the alpha diversity of bacterial communities were analyzed by three-way ANOVA tests. The number and percentage of the unique and shared OTUs among different microhabitats were shown by the Venn diagrams. The Bray–Curtis distance was used to determine the beta diversity of bacterial communities. Non-metric multidimensional scaling analysis (NMDS) and PERMANOVA tests were used to examine the differences in bacterial community compositions across different microhabitats and treatments using the package ‘vegan’ in R. The Student’s T tests were used to further identify the significant differences in the alpha and beta diversity between different microhabitats and treatments. The random forest models were employed to estimate the importance of bacterial OTUs for the variations in the addition of the exogenous *Chlorella pyrenoidosa* using the package ‘randomForest’ in R [29]. We further selected the dominant OTUs from the OTUs identified by the random forest models using the threshold of the average relative abundance of >0.1%, which can be defined as the biomarker for the variations in the *Chlorella pyrenoidosa* addition. The dynamics of relative abundance of these biomarkers among the different treatments in the bacterial communities of water, fish gill, and gut were presented in heatmaps using the package ‘pheatmap’ in R. Canonical correspondence analysis (CCA) was employed to explore the influences of water quality on the bacterial communities in different microhabitats using the package ‘vegan’ in R. Moreover, the command ‘env.fit’ was used to determine whether the impacts of water quality parameters on the bacterial communities were significant.

Topological networks were constructed using the Pearson’s correlation analysis based on the relative abundance of bacterial OTUs in each group to reveal the potential interactions between the bacterial OTUs. To enhance network reliability, we constructed them by selecting bacterial OTUs that had an occurrence of >50% and a relative abundance of >0.01%. Pearson’s correlations having a coefficient of >0.6 or <−0.6 and identified as statistically significant (*p* < 0.05) were included in the construction of the network. Modules were separated from the networks using the fast greedy modularity optimization [30]. To describe the attributes of a network, indices including modularity, clustering coefficient, average path length, network diameter, average degree, and graph density were calculated using the package ‘igraph’ in R [31]. A total of 1000 random networks of equal size were generated by using the ‘igraph’ package in R for each network analysis, and all of the indices of the random networks were calculated individually. A statistical Z test was used to verify whether the network indices between the observed and random networks were significantly different. The networks were visualized by using ‘igraph’ tools in Hiplot Pro (https://hiplot.com.cn/, accessed on 18 October 2022), a comprehensive web service for biomedical data analysis and visualization.

## 3. Results

### 3.1. Effects of the Chlorella pyrenoidosa Addition on Bacterial Community Diversity in Different Microhabitats

**Figure 1:**
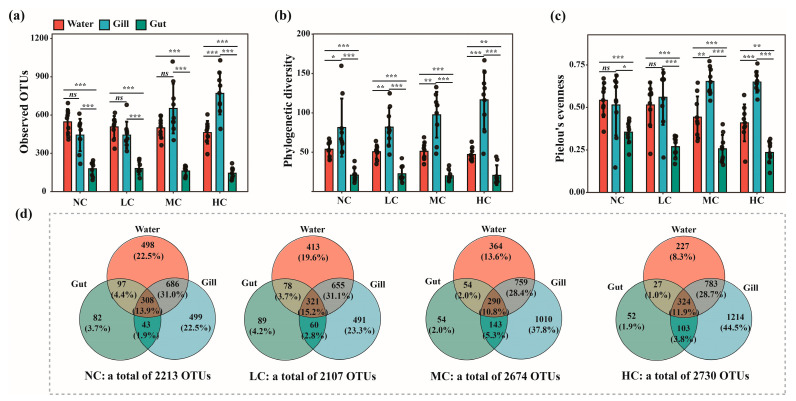
The alpha diversity of bacterial communities inhabiting different microhabitats with different *Chlorella pyrenoidosa* additions. The significant differences in observed OTUs (**a**), phylogenetic diversity (**b**), and Pielou’s evenness (**c**) of bacterial communities between different microhabitats (i.e., water, fish gill, and gut) in treatments with different *Chlorella pyrenoidosa* additions were tested by Student’s T tests. (**d**) shows the number and percentage of shared and unique OTUs in and between bacterial communities inhabiting different microhabitats for treatments with different *Chlorella pyrenoidosa* additions using Venn diagrams. NC, no addition; LC, low concentration; MC, medium concentration; HC, high concentration. ns, *p* > 0.05; * *p* < 0.05; ** *p* < 0.01; *** *p* < 0.001.

The results of three-way ANOVA tests showed that ‘microhabitat’ was the most important factor influencing the richness (observed OTUs), phylogenetic diversity, and evenness (Pielou’s evenness) of bacterial communities, as indicated by much higher F values compared to the ‘treatment’ and ‘time’ factors (Table 1). Moreover, the ‘treatment’ factor showed significant influences on the observed OTUs of bacterial communities (*p* = 0.014), whereas the ‘time’ factor significantly affected the phylogenetic diversity of bacterial communities (*p* < 0.001). Furthermore, the alpha diversity indices were significantly affected by the interactive effects between ‘microhabitat’ and ‘treatment’/’time’ factors (*p* < 0.05). The Student’s T tests showed that the observed OTUs of bacterial communities was significantly lower in fish gut compared to those in water and fish gill (*p* < 0.001), whereas the richness of bacterial communities maintained similar levels between water and fish gill (*p* > 0.05) (Figure S1a). However, significant differences in the phylogenetic diversity and the evenness of bacterial communities were detected among the three microhabitats tested by the paired Student’s T tests (Figure S1b,c). Generally, the bacterial community diversity was found to be the highest in the fish gill, followed by the water, and the lowest was found in the gut. The richness of gill-associated bacterial communities was significantly higher in the MC and HC treatments compared to those in the NC and LC treatments (Figure 1a). We also found that the phylogenetic diversity and evenness of gill-associated bacterial communities were higher in the MC and HC treatments compared to those in the NC and LC treatments, although no significant differences in phylogenetic diversity and evenness of bacterial communities were detected between treatments in fish gill (Figure 1b,c). Furthermore, the decreasing patterns were observed in the alpha diversity of bacterial communities in water and gut alongside the increasing *Chlorella pyrenoidosa* addition, especially for the phylogenetic diversity of bacterial communities in fish gut.

The Venn diagrams showed that the overall number of bacterial OTUs within all microhabitats increased in the MC and HC treatments compared to those in the NC and LC treatments (Figure 1d. Much higher numbers of the unique bacterial OTUs associated with the fish gill than in the water and gut were observed in the MC and HC treatments (Figure 1d). Moreover, the *Chlorella pyrenoidosa* addition increased the numbers and proportions of unique OTUs of the gill-associated bacterial communities, whereas the numbers and proportions of the unique bacterial OTUs in the water and gut decreased. It was also detected that numbers and proportions of the shared bacterial OTUs between the water and gill were much higher than those between fish gut and water column/fish gill in all treatments.

### 3.2. The Bacterial Community Structure of Different Microhabitats Affected by the Chlorella pyrenoidosa Addition

**Figure 2:**
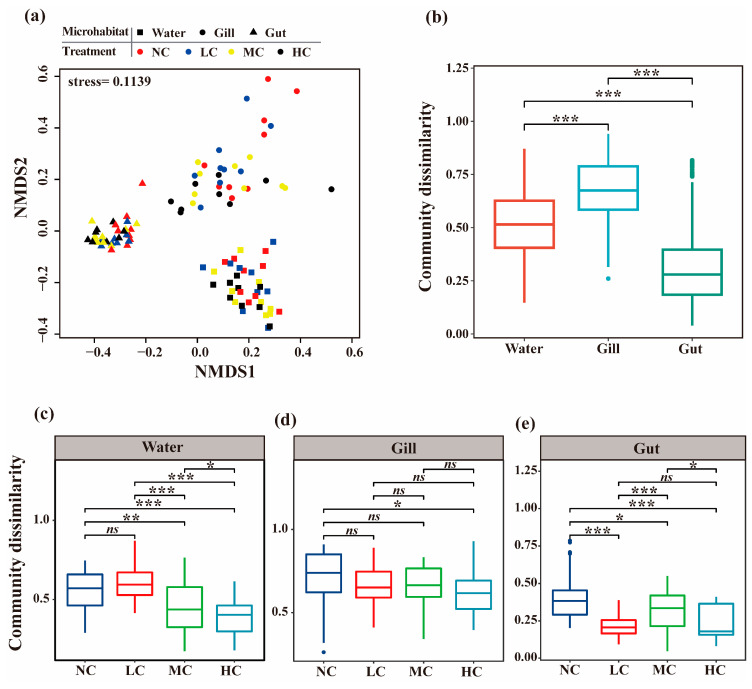
The community dissimilarity (beta diversity) of bacterial communities inhabiting different microhabitats (water, gill, and gut) with different *Chlorella pyrenoidosa* additions based on Bray–Curtis distance. (**a**) Non-metric multidimensional scaling (NMDS) analysis showing the community dissimilarity of bacterial communities. (**b**) The comparisons of bacterial dissimilarity between different microhabitats; the samples from different treatments and sampling timepoints were mixed. (**c**–**e**) The comparisons of bacterial dissimilarity between different treatments (different *Chlorella pyrenoidosa* additions) in water, fish gill, and gut; the samples from different sampling timepoints were mixed. NC, no addition; LC, low concentration; MC, medium concentration; HC, high concentration. Significant differences in community dissimilarity were tested by Student’s T tests. ns, *p* > 0.05; * *p* < 0.05; ** *p* < 0.01; *** *p* < 0.001.

The result of the PERMANOVA test indicated that the bacterial community compositions were significantly different among different microhabitats, treatments, and sampling timepoints (Table 2). However, the ‘microhabitat’ factor (R^2^ = 0.53) was responsible for a much higher explanation for the community dissimilarity compared to the ‘treatment’ (R^2^ = 0.03) and ‘time’ (R^2^ = 0.05) factors. Furthermore, the ‘microhabitat’ factor interacting with ‘treatment’/’time’ factors has significant influences on the bacterial community compositions, although their explanations were quite low. The NMDS diagram also showed that the samples belonging to the same microhabitat were clustered together, whereas those samples from different microhabitats were distinctly separated (Figure 2a). However, the community dissimilarity between different treatments and timepoints was rarely distinguished. The comparisons of beta diversity of bacterial communities inhabiting different microhabitats demonstrated that the beta diversity of the bacterial community associated with the fish gill was significantly higher than that in water and gut (*p* < 0.001) (Figure 2b). Moreover, the beta diversity of the bacterial community in water was significantly higher than that in fish gut (*p* < 0.001). The bacterial community dissimilarities were distinctly influenced by the *Chlorella pyrenoidosa* addition between the different microhabitats (Figure 2c–e). In the water column, the bacterial communities of the NC and LC treatments maintained a similar level of dissimilarities within the community, which were significantly higher than those of the MC and HC treatments (*p* < 0.01) (Figure 2c). On the contrary, there were no significant differences in the dissimilarities of gill-associated bacterial communities among the NC, LC, and MC treatments (*p* > 0.05). However, the HC treatment showed significantly lower bacterial community dissimilarity than the NC treatment (*p* < 0.05) (Figure 2d). Furthermore, the *Chlorella pyrenoidosa* addition significantly decreased the bacterial community dissimilarities of fish gut, which was confirmed by the significantly higher bacterial community dissimilarities of the NC treatment compared to the other treatments (*p* < 0.05) (Figure 2e).

### 3.3. The Taxonomic Compositions of Bacterial Communities in Different Microhabitats Affected by the Chlorella pyrenoidosa Addition

The average relative abundance of nine bacterial phyla was found to exceed 0.5%, and were identified as the dominant phyla in bacterial communities across different microhabitats and treatments. However, the relative abundance of these dominant bacterial phyla showed notable differences between different microhabitats and treatments, as shown in Figure S3. Proteobacteria was the predominant bacterial phylum in the water column, comprising over 60% of the total relative abundance. Moreover, it was observed that the abundance of Proteobacteria improved with an increase in the *Chlorella pyrenoidosa* addition. Actinobacteriota and Bacteroidota were also dominant in the bacterial communities of the water column, with average relative abundances of >10%. Increasing the *Chlorella pyrenoidosa* addition was found to evidently increase the relative abundance of Actinobacteriota, whereas it caused a reduction in the relative abundance of Bacteroidota. In fish gill, Proteobacteria was also found to be the most abundant bacterial phylum (with an average relative abundance above 50%), followed by Actinobacteria (with an average relative abundance above 15%) and then Fusobacteriota (with an average relative abundance of approximately 8%). Furthermore, increasing the *Chlorella pyrenoidosa* addition resulted in decreased relative abundances of Proteobacteria and Fusobacteriota, while increasing the relative abundance of Actinobacteriota. Actinobacteriota showed overwhelmingly high relative abundance, with a relative abundance of > 85% in the bacterial communities of fish gut, and the relative abundance increased with the increasing *Chlorella pyrenoidosa* addition. Moreover, Fusobacteriota and Proteobacteria were also dominant in the bacterial communities of fish gut, with relative abundances of around 5%, and they both showed lower relative abundances in the MC and HC treatments compared to those in the NC and LC treatments. In addition, we analyzed the variations in the relative abundance of dominant OTUs (with a relative abundance of >0.5%) inhabiting the three microhabitats across different treatments (Figure S4). A total of 29, 24, and 14 OTUs were identified as the dominant OTUs in the bacterial communities of water, fish gill, and fish gut, respectively. Generally, more than 75% (22/29) of the dominant OTUs of the bacterial communities of water showed a decrease in the relative abundance in the HC treatment compared to those in the NC treatment (Figure S4a). Similarly, approximately 65% (9/14) of the dominant OTUs of bacterial communities inhabiting the fish gut exhibited a decline in relative abundance during the HC treatment compared to the NC treatment (Figure S4c). On the contrary, more than 50% (13/24) of the dominant OTUs of bacterial communities associated with the fish gill showed higher relative abundance during the HC treatment comparing with the NC treatment (Figure S4b).

**Figure 3:**
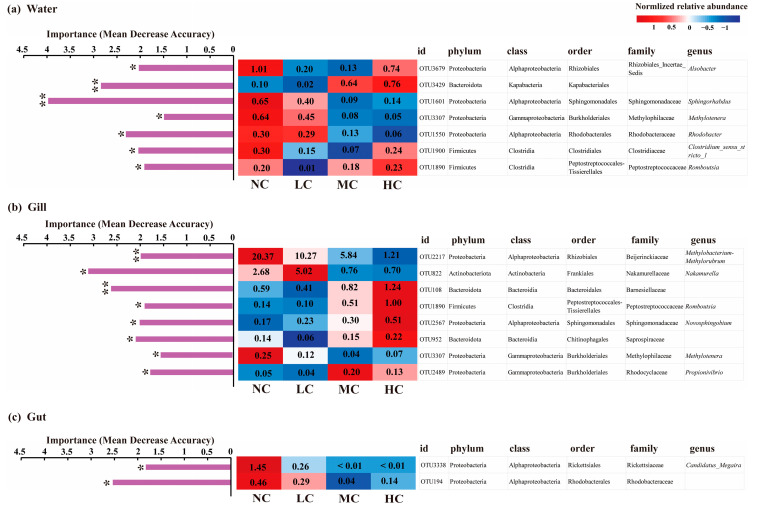
Bacterial taxa with mean relative abundance > 0.1% that were significantly influenced by the *Chlorella pyrenoidosa* addition in water (**a**), fish gill (**b**), and gut (**c**) detected by random forest models. The bar charts on the left show the importance of these bacterial taxa on the bacterial community dissimilarity among different treatments in each microhabitat using the index of mean decrease accuracy. The heatmaps on the right show the normalized relative abundance (by rows) of each bacterial OTU in different treatments. The numbers in the color blocks represent the non-normalized relative abundance (%). NC, no addition; LC, low concentration; MC, medium concentration; HC, high concentration. * *p* < 0.05; ** *p* < 0.01.

We employed random forest models to identify the bacterial OTUs (biomarkers) making significant contributions to bacterial community dissimilarity across different treatments in each microhabitat (Figure S5). The results of random forest models showed that the fish gill-associated microbiome contained the greatest number of biomarkers, followed by water, and the fish gut had the fewest. We further selected biomarkers with a relative abundance of >0.1%. We obtained seven, eight, and two abundant biomarkers in the bacterial communities of water, fish gill, and gut, respectively (Figure 3). In water, OTU3429, which was assigned to the order Kapabacteriales, phylum Bacteroidota, showed a higher relative abundance in the MC and HC treatments compared to the NC and LC treatments, whereas the other six biomarkers showed contrasting patterns. However, five biomarkers (OTU108, OTU1890, OTU2567, OTU952, and OTU2489) with the relative abundance of >0.1% identified from the gill-associated bacterial communities displayed an increase trend in relative abundance with the *Chlorella pyrenoidosa* addition. Another three biomarkers (OTU2217, OTU822, and OTU3307) generally showed a decline in the relative abundance with the *Chlorella pyrenoidosa* addition. Among these biomarkers in fish gill, the relative abundance of OTU2217, which belonged to the genus *Methylobacterium-Methylorubrum* of class Alphaproteobacteria, varied from 20.37% in the NC treatment to 1.21% in the HC treatment. Similarly, OTU822, which was assigned to the genus *Nakamurella* of phylum Actinobacteriota, showed high variance in the relative abundance among treatments. Furthermore, both OTU108 and OTU952, which belonged to the families Barnesiellaceae (phylum Bacteroidota) and the genera *Romboutsia* (phylum Firmicutes), respectively, were dominant in the gill-associated bacterial communities in the HC treatment, with the relative abundance of >1%. In the fish gut, the two biomarkers decreased their relative abundances with the *Chlorella pyrenoidosa* addition. Moreover, OTU3307 and OTU1890 were identified as biomarkers in the bacterial communities of both water and gill. OTU3307 showed a trend of decreasing relative abundance with the increasing addition of exogenous *Chlorella pyrenoidosa* in both water and gill. However, OTU1890 exhibited an increasing trend in relative abundance with the increasing *Chlorella pyrenoidosa* addition in the fish gill. In water, the relative abundance of OTU1890 was evidently lower in the LC treatment compared with those in the other three treatments.

### 3.4. The Relationships between the Water Quality and Bacterial Communities in Different Microhabitats

**Figure 4:**
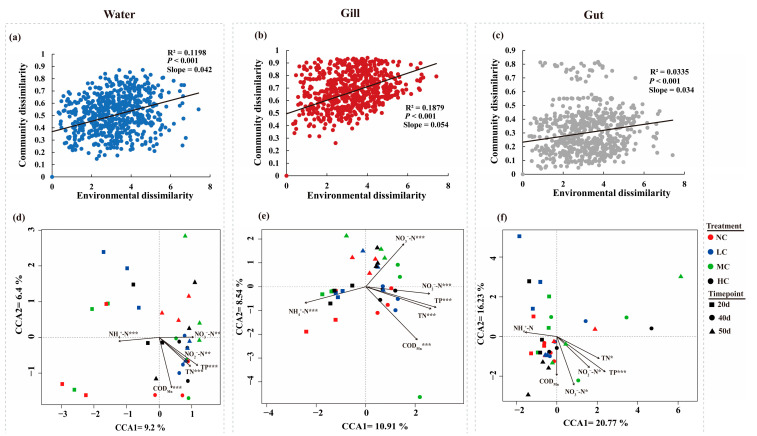
The influences of the water quality on the bacterial communities in different microhabitats; (**a**–**c**) The linear regressions of the bacterial community dissimilarity (based on Bray–Curtis distance) against the environmental dissimilarity (based on Euclidean distance, including all the water quality parameters involved in this study); (**d**–**f**) Canonical correspondence analyses (CCA) showing the explanation of the water quality parameters for the shift in bacterial communities. TN, total nitrogen; TP, total phosphorus; COD_Mn_, chemical oxygen demand; NH_4_^+^-N, ammonia nitrogen; NO_3_^−^-N, nitrate nitrogen; NO_2_^−^-N, nitrite nitrogen. * *p* < 0.05; ** *p* < 0.01; *** *p* < 0.001.

The linear regression analyses showed that the bacterial community dissimilarities of water, fish gill, and gut were all significantly correlated with the environmental dissimilarities (*p* < 0.001; Figure 4a–c). Moreover, the significant relationship between the gill-associated bacterial community dissimilarity and the environmental dissimilarity was stronger than those of water and gut, indicated by a higher R^2^ value. Moreover, the slope of the relationship between the gill-associated bacterial community dissimilarity and the environmental dissimilarity was also steeper than those of water and gut. Nonetheless, the R^2^ values of all the linear regressions were quite low (<0.5). The CCA further explored the influences of these water quality parameters on the bacterial communities. Although the variations in bacterial communities of all the three microhabitats explained by the water quality parameters were quite low, as indicated by the low explanation of the CCA axes, the contributions of TN, TP, COD_Mn_, NH_4_^+^-N, NO_3_^−^-N, and NO_2_^−^-N to the shift of bacterial communities were different among the water, fish gill, and gut (Figure 4d–f). All of the water quality parameters were significantly correlated with the variations in the bacterial communities in water and fish gill. However, TN, TP, NO_3_^−^-N, and NO_2_^−^-N were significantly correlated with the variations in the bacterial communities in fish gut, whereas COD_Mn_ and NH_4_^+^-N were not.

### 3.5. The Co-Occurrence Patterns of Bacterial Communities in Different Microhabitats Influenced by the Chlorella pyrenoidosa Addition

**Figure 5:**
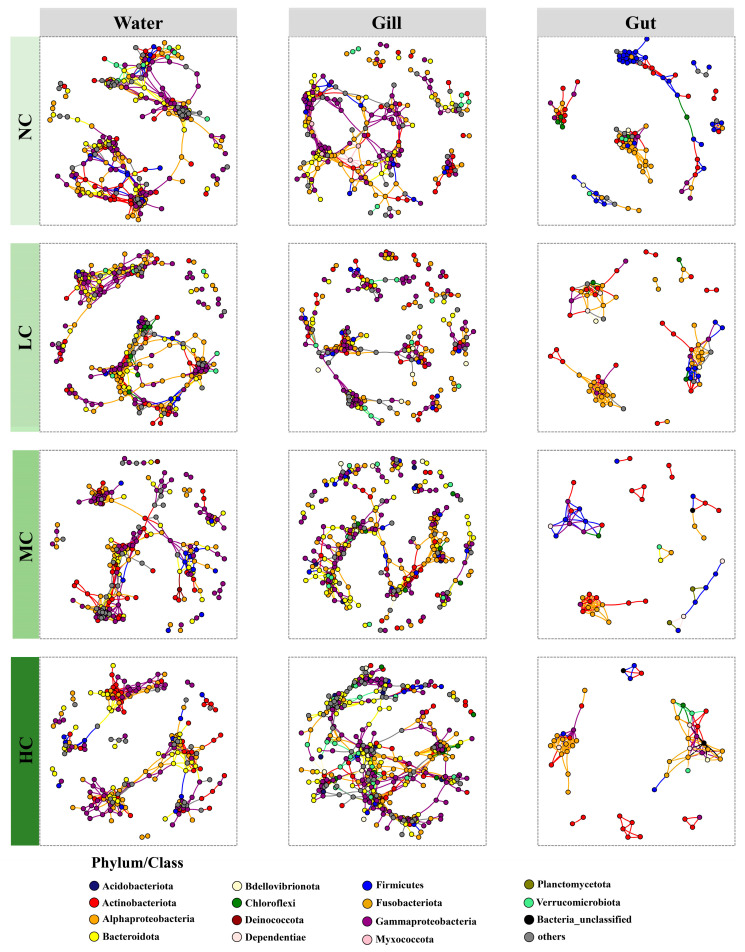
The bacterial community networks in water, fish gill, and gut with different *Chlorella pyrenoidosa* additions. NC, no addition; LC, low concentration; MC, medium concentration; HC, high concentration.

A total of 12 bacterial community networks were constructed to explore the variations in the co-occurrence patterns of bacterial communities inhabiting different microhabitats with different *Chlorella pyrenoidosa* additions (Figure 5). The results of the Z tests for comparing the random and observed networks showed that all the bacterial networks were non-random, and the topological parameters were significantly different from those of random networks (*p* < 0.05; Table 3). With no addition of the exogenous *Chlorella pyrenoidosa*, the bacterial community network of the water harbored the highest number of network nodes, followed by the bacterial community network of the fish gill, whereas the bacterial community network of the fish gut possessed the fewest number of network nodes (Table 3). Moreover, the average degree of the bacterial community network was much higher in water than those in the fish gill and gut during the NC treatment. As the exogenous *Chlorella pyrenoidosa* was added into the aquaculture systems, a decreasing pattern in the number of the nodes and links was observed in the bacterial community networks in water. Similarly, the average degree, average path length, network diameter, and assortativity of the bacterial community networks in the water all decreased during the treatments with the *Chlorella pyrenoidosa* addition compared to the NC treatment. However, the modularity of the bacterial community network in the water was much higher in the HC treatment compared to the NC, LC, and MC treatments. The topological parameters of bacterial community networks associated with the fish gill showed distinct dynamics with the *Chlorella pyrenoidosa* addition from those of bacterial community networks in water. Specifically, more nodes and links were involved in the bacterial community networks associated with the fish gill when the addition of exogenous *Chlorella pyrenoidosa* increased. The average degree of the bacterial community network associated with the fish gill was much higher in the HC treatment compared to the other three treatments. Furthermore, we found that the average path length and network diameter was obviously higher in the MC and HC treatments than in the NC and LC treatments for the bacterial community networks associated with the fish gill. In the fish gut, the number of network nodes and links declined in the treatments with the addition of exogenous *Chlorella pyrenoidosa* compared to the NC treatment. The average degree, average path length, and network diameter of the bacterial community networks also decreased during the treatments with the *Chlorella pyrenoidosa* addition compared to the NC treatment.

## 4. Discussion

An increasing trend of alpha diversity of gill-associated bacterial communities was observed in response to the increasing *Chlorella pyrenoidosa* addition. Similarly, the number and percentage of the unique OTUs increased in the gill-associated bacterial communities along with the *Chlorella pyrenoidosa* addition. Furthermore, more than half of the dominant OTUs of the gill-associated bacterial communities increased their relative abundance with the *Chlorella pyrenoidosa* addition. These results suggested that the presence of *Chlorella pyrenoidosa* could be beneficial for increasing the diversity of bacterial communities associated with the fish gill through simultaneously promoting more new colonizers and stimulating the enrichment of the dominant local taxa on the fish gill. The fish gill act as primary sites for gas and waste exchange in fishes, as well as for mucosal immune interactions, osmoregulation, and detoxification [32], which is potentially influenced by high local concentrations of oxygen and nutrients [33]. The presence of *Chlorella pyrenoidosa* could affect the fish gill through absorbing ammonia and producing oxygen, thus contributing to the variations in the gill-associated bacterial diversity. However, it seemed that the addition of exogenous *Chlorella pyrenoidosa* showed minor impacts on the alpha diversity of bacterial communities in the fish gut.

The fish microbiota showed significant differences in the community structures and compositions from those in the water column, which were consistent with previous studies [20,34]. Furthermore, many studies have reported that the fish microbiome exhibits tissue specificity [35,36]. It has been discussed that the microbes can colonize the fish body niches from the species pools of the external community through niche-based selection processes [37]. However, compared to the fish gut, the fish gill can have more frequent exchange of water and gas with the water environment [38], which would result in similar dominant phyla shared by the water and gill-associated samples. In this study, Proteobacteria and Actinobacteriota dominated in the bacterial communities in both the water column and fish gill. Furthermore, Actinobacteriota showed an increasing pattern with the *Chlorella pyrenoidosa* addition in both the water column and the fish gill. Bacteria taxa belonging to the phylum Actinobacteriota were ubiquitous in the aquaculture waters as well as in the fish gill [39,40,41], which was reported to display a symbiotic relationship with the genus *Chlorella* [42]. Moreover, Fusobacteriota were found to be the abundant bacterial phyla in the fish microbiota. Fusobacteriota has been identified as the core taxon of bacterial communities associated with the skin, mucosa, and gut in many fish [21,43,44]. Moreover, Fusobacteriota could be indicative of the health of the fish; for example, Yang et al. (2023) found that Fusobacteriota was dominant in the gut-associated bacterial communities of the healthy yellow catfish rather than those of diseased fish infected by Edwardsiella ictalurid [45]. Guo et al. (2023) demonstrated that the relative abundance of Fusobacteriota decreased in the gut-associated bacterial communities of Luciobarbus capito in response to the thermal stress [44]. Here, we observed a decline in the relative abundance of Fusobacteriota in the gut-associated bacterial communities with addition of the exogenous *Chlorella pyrenoidosa*; however, whether the decline in the relative abundance of Fusobacteriota suggested a health risk for the cultured fish still needs to be further explored.

At a lower taxonomic level, many more bacterial taxa associated with the fish gill showed significant responses in the relative abundance to the *Chlorella pyrenoidosa* addition than those inhabiting the water and gut, which suggested higher sensitivity of gill-associated bacterial communities than the water- and gut-associated bacterial communities. Furthermore, a higher R^2^ value and slope of the linear regressions on the gill-associated bacterial community dissimilarity against the environmental dissimilarity, compared to those of the water and the fish gut, also indicated that changes in the water quality can induce a higher shift in the gill-associated bacterial communities. On one hand, the mucosal surface of the fish gill represented an important barrier that supported and regulated a wide variety of microbial communities, which were essential for fish health [15]. On the other hand, the bacterial communities attached to these mucosal surfaces would be susceptible to surrounding environmental changes. For instance, Huang et al. (2022) found that the bacterial communities associated with the fish gill and skin showed higher variety in diversity and composition in response to exposure to microplastics in comparison with those in gut-associated bacterial communities [46]. In the gill-associated bacterial communities, bacterial taxa assigned to the genus *Methylobacterium-Methylorubrum* of class Alphaproteobacteria declined in their relative abundance by more than 90% with addition of high concentrations of *Chlorella pyrenoidosa*. Bacteria belonging to the genus *Methylobacterium-Methylorubrum*, which are known to be facultative methylotrophs, are phylogenetically related to *Methylobacterium* [47]. These bacterial taxa are able to colonize different surfaces and can adhere and form biofilm [48]. Furthermore, *Methylobacterium* is potentially important for protecting the host against pathogens in fish skin by producing poly-β-hydroxybutyrate and degrading short-chain fatty acids [49,50,51]. In the present study, whether the fading of *Methylobacterium-Methylorubrum* bacteria with the *Chlorella pyrenoidosa* addition affected the immune-related functions of the mucosa of fish gill was still unclear. Moreover, the bacterial taxon belonging to the family Barnesiellaceae showed an increasing pattern in the relative abundance in response to the increasing *Chlorella pyrenoidosa* addition. Bacterial taxa assigned to the family Barnesiellaceae have been observed in the gut microbiomes of fish, but they were rarely detected in the fish gill. A study investigating the response of fish gut-associated bacterial communities to transport stress found that the taxa assigned to the family Barnesiellaceae decreased their relative abundance in response to the transport stress, which suggested the depletion of Barnesiellaceae were associated with the stress [52]. On the contrary, the enrichment of Barnesiellaceae bacteria in the HC treatment might indicate a healthy state of fish with the *Chlorella pyrenoidosa* addition. Overall, compared to the water column and gut, more bacterial taxa of the gill-associated bacterial communities showed significant responses to the *Chlorella pyrenoidosa* addition. These biomarkers showing significant responses to environmental changes might have important influences on fish mucosal immunity, which still requires further exploration to explain their causal relationships.

Although it has been reported that aquaculture activities would reduce the complexity of planktonic microbial community networks in comparison with non-artificial ecosystems [39,53], changes in the topological parameters of bacterial community networks in the three microhabitats with the *Chlorella pyrenoidosa* addition could imply variations in the potential community functions. Distinct variations in the topological networks of bacterial communities in response to the *Chlorella pyrenoidosa* addition were observed in water, fish gill, and gut. As the *Chlorella pyrenoidosa* were added into the aquaculture systems, the topological network of bacterial communities was smaller in size and less complex in water and gut, as indicated by fewer network nodes, lower average degree, shorter average path length, and lower network diameter. However, the network size and complexity of bacterial communities associated with the fish gill were higher with the *Chlorella pyrenoidosa* addition. A study focusing on the co-occurrence patterns of microbial communities in the water columns of two different lake zones showed that the aquatic microbial community network’s complexity was lower in the algae-dominated environment than that in macrophyte-dominated environment [54], which was similar to our results. Furthermore, despite limited research on the impact of algae addition on the gut-associated microbiota of fish, there is also a lack of understanding regarding the influence of algae addition on the gut-associated microbial community network. However, a field investigation study on the gut microbiota of fish suggests that diet is a major driving factor in shaping the composition and co-occurrence patterns of the gut microbiota, even within the same lake [55]. Therefore, the addition of exogenous *Chlorella pyrenoidosa* might drive changes in the co-occurrence patterns of the gut microbiota by altering the diet of tilapia. Moreover, as predicted by the theoretical work in ecology, the complexity of microbial community networks could suggest the resistance of microbial communities to disturbances [41,56]. It has been also proposed that a more complex network of species interactions may buffer response to environmental changes [57]. Thus, the increasing complexity of gill-associated bacterial communities in response to the *Chlorella pyrenoidosa* addition could imply the high resistance of fish gill to environmental and biological disturbances, but their mechanisms should be further explored.

## 5. Conclusions

In summary, our results showed that *Chlorella pyrenoidosa* addition exhibited distinct influences on the bacterial communities among the water, fish gill, and gut. As the *Chlorella pyrenoidosa* addition increased, the number of unique OTUs in the gill-associated bacterial communities greatly increased, leading to a noticeable increase in the richness of gill-associated bacterial communities. However, bacterial diversity in the water column and gut were not significantly affected by the *Chlorella pyrenoidosa* addition. The bacterial community compositions were significantly different among the three microhabitats. Furthermore, random forest models detected different biomarkers in response to the *Chlorella pyrenoidosa* addition among the bacterial communities of water, fish gill, and gut. The gill-associated bacterial communities harbored a higher number of biomarkers compared to the water samples, whereas the intestinal bacterial communities had the lowest number of biomarkers. Moreover, the network analyses revealed that the *Chlorella pyrenoidosa* addition led to a reduction in network complexity within the bacterial communities of both the water and the gut; however, it slightly increased the network complexity in the gill-associated bacterial communities. Overall, the greatest impact of the *Chlorella pyrenoidosa* addition was observed in the gill-associated bacterial community. The increased bacterial diversity, shifts in community composition, and enhanced network complexity resulting from the *Chlorella pyrenoidosa* addition could have implications for the immune function of the mucosa of tilapia gill. Although water quality showed significant impacts on these bacterial communities, the explanation of water quality for the shift of bacterial communities in different microhabitats was quite low. The unknow mechanisms underlying the assembly of bacterial communities, as well as the causal relationships of the microbial diversity and taxonomy with the water quality and fish health during the *Chlorella pyrenoidosa* addition, still need to be further explored.

## Figures and Tables

**Table 1:** The effects of microhabitats (water, gill, and gut), treatments (different *Chlorella pyrenoidosa* additions), and time (sampling timepoints) on the alpha diversity indexes of bacterial communities using three-way ANOVA tests.

	Observed OTUs		Phylogenetic Diversity		Pielou’s Evenness	
	F	*p*	F	*p*	F	*p*
Microhabitat	**160.5**	**<0.001**	**224.9**	**<0.001**	**86.6**	**<0.001**
Treatment	**3.8**	**0.014**	2.6	>0.05	0.7	>0.05
Time	0.7	>0.05	**15.1**	**<0.001**	0.5	>0.05
Microhabitat × Treatment	**9.6**	**<0.001**	**4.7**	**<0.001**	**4.5**	**<0.001**
Microhabitat × Time	**3.2**	**0.018**	**11.8**	**<0.001**	**6.3**	**<0.001**
Treatment × Time	0.6	>0.05	**2.5**	0.033	0.9	>0.05
Microhabitat × Treatment × Time	0.7	>0.05	1.6	>0.05	0.3	>0.05

**Table 2:** PERMANOVA test on the significant differences in community compositions between different microhabitats (water, gill, and gut), treatments (different *Chlorella pyrenoidosa* additions), and sampling timepoints based on the Bray–Curtis distance.

	Df	Sum of Sqs	R^2^	F	*p* (>F)
**Microhabitat**	**2**	**17.4**	**0.53**	**81.5**	**0.001**
**Treatment**	**3**	**0.9**	**0.03**	**2.7**	**0.002**
**Time**	**2**	**1.5**	**0.05**	**7.1**	**0.001**
**Microhabitat × Treatment**	**6**	**1.3**	**0.04**	**2.0**	**0.004**
**Microhabitat × Time**	**4**	**2.1**	**0.06**	**5.0**	**0.001**
Treatment × Time	6	0.9	0.03	1.3	0.118
Microhabitat × Treatment × Time	12	1.3	0.04	1.0	0.445
Residual	72	7.7	0.23		
Total	107	33.0	1.00		

**Table 3:** The topological parameters of bacterial community networks in different microhabitats with different *Chlorella pyrenoidosa* additions. NC, no addition; LC, low concentration; MC, medium concentration; HC, high concentration. Significant differences in the transitivity, average path length, network diameter, modularity, and assortativity between the observed network and the random networks were tested by Z tests. *** *p* < 0.001.

	Water			
	NC	LC	MC	HC
**No. of nodes**	277	256	223	214
**No. of edges**	2182	1309	1216	870
**Average degree**	15.8	10.2	10.9	8.1
**Graph density**	0.06	0.04	0.05	0.04
**Transitivity**	0.81 ***	0.70 ***	0.78 ***	0.70 ***
**Average path length**	7.23 ***	4.61 ***	4.16 ***	5.13 ***
**Network diameter**	20.3 ***	10.0 ***	10.8 ***	14.6 ***
**Modularity**	0.67 ***	0.71 ***	0.69 ***	0.77 ***
**Assortativity**	0.81 ***	0.62 ***	0.76 ***	0.59 ***
	**Gill**			
	**NC**	**LC**	**MC**	**HC**
**No. of nodes**	217	233	286	413
**No. of edges**	762	981	982	2777
**Average degree**	7.0	8.4	6.9	13.4
**Graph density**	0.03	0.04	0.02	0.03
**Transitivity**	0.66 ***	0.74 ***	0.64 ***	0.73 ***
**Average path length**	4.95 ***	3.72 ***	8.22 ***	7.27 ***
**Network diameter**	11.9 ***	11.1 ***	21.1 ***	18.0 ***
**Modularity**	0.76 ***	0.77 ***	0.80 ***	0.74 ***
**Assortativity**	0.66 ***	0.72 ***	0.58 ***	0.73 ***
	**Gut**			
	**NC**	**LC**	**MC**	**HC**
**No. of nodes**	107	75	49	53
**No. of edges**	480	240	101	164
**Average degree**	9.0	6.4	4.1	6.2
**Graph density**	0.08	0.09	0.09	0.12
**Transitivity**	0.79 ***	0.71 ***	0.83 ***	0.78 ***
**Average path length**	2.92 ***	2.01 ***	1.73 ***	1.90 ***
**Network diameter**	9.7 ***	4.6	4.6	4.5
**Modularity**	0.65 ***	0.68 ***	0.66 ***	0.59 ***
**Assortativity**	0.71 ***	0.44 ***	0.74 ***	0.64 ***

## Data Availability

All the data involved in this research were submitted to the NCBI Sequence Read Archive (SRA) database (Bioproject ID: PRJNA997058).

## Supplementary Materials

The following supporting information can be downloaded at: https://www.mdpi.com/article/10.3390/biology12091209/s1, Figure S1: The comparisons in alpha diversity of bacterial communities in water, fish gill, and gut [58,59,60,61]. The samples from different treatments and timepoints were mixed. The significant differences in observed OTUs (a), phylogenetic diversity (b), and Pielou’s evenness (c) of bacterial communities between different microhabitats (i.e., water, gill, and gut) were tested by Sstudent’s Tt tests. ns, *p* > 0.05; * *p* < 0.05; ** *p* < 0.01; *** *p* < 0.001; Figure S2: The comparisons of alpha diversity of bacterial communities between different treatments (different *Chlorella pyrenoidosa* additions) in water, fish gill, and gut. The samples from different timepoints were mixed. The significant differences in observed OTUs (a), phylogenetic diversity (b), and Pielou’s evenness (c) of bacterial communities between different microhabitats (i.e., water, gill, and gut) indicated by different letters were tested by ANOVA tests followed by Turkey post hoc tests; Figure S3: The relative abundance of the dominant phyla (relative abundance > 0.5%) of bacterial communities inhabiting different microhabitats in treatments with different *Chlorella pyrenoidosa* additions. NC, no addition; LC, low concentration; MC, medium concentration; HC, high concentration; Figure S4: Heatmaps showing the relative abundance of dominant OTUs (mean relative abundance > 0.5%) across different treatments (different *Chlorella pyrenoidosa* additions) in water (a), gill (b), and gut (c). Figure S5: Bacterial taxa that are significantly influenced by the *Chlorella pyrenoidosa* addition in water, gill, and gut, as detected by random forest models.
