# Supplementary material for: The Gill-Associated Bacterial Community Is More Affected by Exogenous Chlorella pyrenoidosa Addition than the Bacterial Communities of Water and Fish Gut in GIFT Tilapia (Oreochromis niloticus) Aquaculture System
Source: Biology (Basel). 2023 Sep 5;12(9):1209. doi: 10.3390/biology12091209 (PMC10525419; doi:10.3390/biology12091209)
Supplement: Supplementary file 1 [file biology-12-01209-s001.zip › biology-2553956-supplementary.pdf]

Supplementary Materials

Title: The gill-associated microbiome is more affected by exogenous *Chlorella pyrenoidosa* addition than the microbiomes in the water and gut in the GIFT Tilapia (*Oreochromis niloticus*) aquaculture system

Shunlong Meng[1, 2†], Huimin Xu[1†], Lu Qin[2†], Xi Chen[1], Liping Qiu[1], Dandan Li[1], Chao Song[1,2], Limin Fan[1,2], Gengdong Hu[1], Pao Xu[1,2]

[1] Freshwater Fisheries Research Center, Chinese Academy of Fishery Sciences; Scientific Observing and Experimental Station of Fishery Resources and Environment in the Lower Reaches of the Yangtze River. Wuxi 214081. China

[2] Wuxi Fishery College, Nanjing Agricultural University, Wuxi 214081, China

[†] These authors contributed equally to this work and should be considered co-first authors.

* Correspondence: xup@ffrc.cn (P.X.)

Methods and materials

Bioinformatics

The paired-end raw sequences were processed with QIIME v1.9.1[1]. Firstly, we filtered sequences for all fastq files using the following criteria: (i) sequences having an average quality score < 25 and a read length < 200 bp were removed; (ii) reads having any mismatch with primer matching or comprising blurred characters were discarded; and (iii) sequences that could not be assembled were removed. The filtering on sequences was conducted using the command '*split_libraries_fastq.py*' and '*join_paired_ends.py*' of QIIME. UCHIME de novo strategy was used for identifying and removing chimeric sequences [2]. We then clustered operational taxonomic units (OTUs) using UCLUST with a 97% identity threshold [3]. The taxonomic information of each OTU was then explored based on SILVA database (Release138 http://www.arb-silva.de). Non-bacterial sequences were also removed by the command '*filter_taxa_from_otu_table.py*' for the subsequent analyses. To minimize sequencing errors, OTUs having reads < 15 (0.0005% of total reads) were discarded using the command '*filter_otus_from_otu_table.py*' [4]. Finally, samples were rarefied at 24,577 sequences according to the minimum sequence number.

References:

1. Caporaso, J.G.; Lauber, C.L.; Walters, W.A.; Berglyons, D.; Huntley, J.; Fierer, N.; Owens, S.M.; Betley, J.; Fraser, L.; Bauer, M. Ultra-high-throughput microbial community analysis on the Illumina HiSeq and MiSeq platforms. *The ISME Journal* **2012**, *6*, 1621-1624.
2. Edgar, R.C.; Haas, B.J.; Clemente, J.C.; Quince, C.; Knight, R. UCHIME improves sensitivity and speed of chimera detection. *Bioinformatics* **2011**, *27*, 2194-2200, doi:10.1093/bioinformatics/btr381.
3. Edgar, R.C. Search and clustering orders of magnitude faster than BLAST. *Bioinformatics* **2010**, *26*, 2460-2461, doi:10.1093/bioinformatics/btq461.
4. Bokulich, N.A.; Subramanian, S.; Faith, J.J.; Gevers, D.; Gordon, J.I.; Knight, R.; Mills, D.A.; Caporaso, J.G. Quality-filtering vastly improves diversity estimates from Illumina amplicon sequencing. *Nature Methods* **2013**, *10*, 57-U11, doi:10.1038/nmeth.2276.

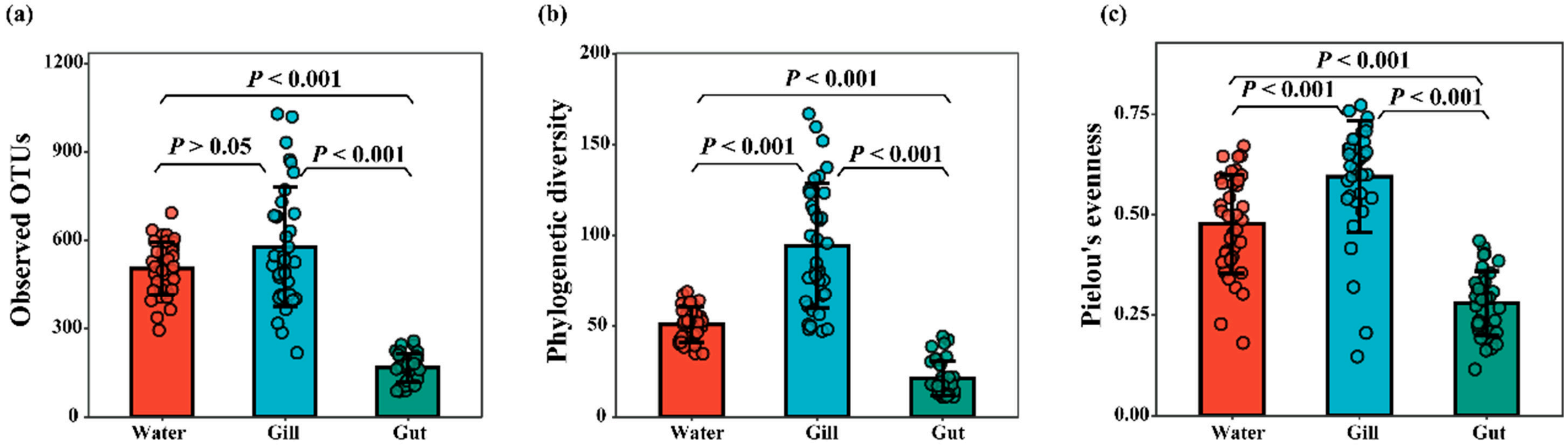

Figure S1. The comparisons in alpha diversity of bacterial communities in water, gill and gut. The samples from different treatments and timepoints were mixed. The significant differences in observed OTUs (a), phylogenetic diversity (b), and Pielou's evenness (c) of bacterial communities between different microhabitats (i.e., water, gill and gut) were tested by student's t tests. ns, $p > 0.05$; * $p < 0.05$; ** $p < 0.01$; *** $p < 0.001$.

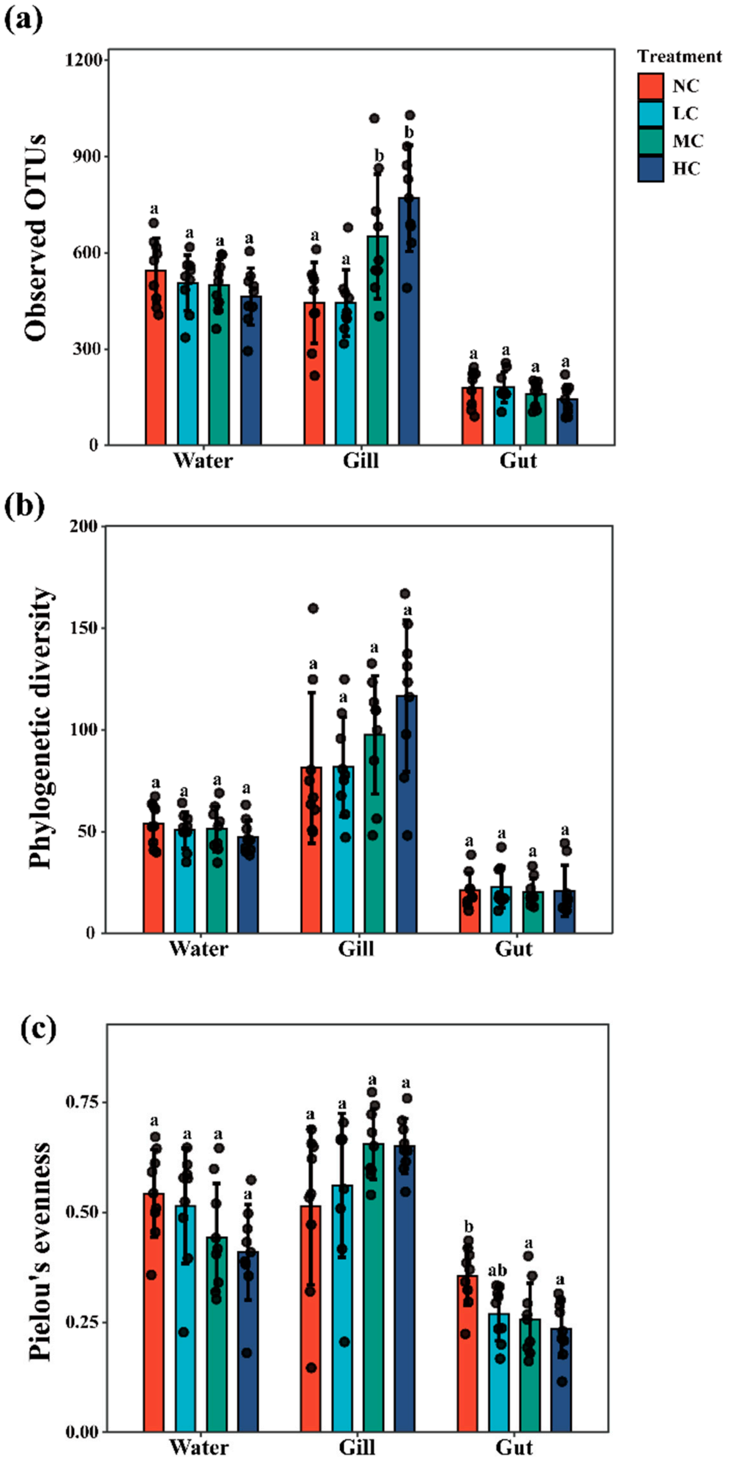

Figure S2. The comparisons of alpha diversity of bacterial communities between different treatments (different *Chlorella pyrenoidosa* additions) in water, gill and gut. The samples from different timepoints were mixed. The significant differences in observed OTUs (a), phylogenetic diversity (b), and Pielou's evenness (c) of bacterial communities between different microhabitats (i.e., water, gill and gut) indicated by different letters were tested by ANOVA tests followed by Turkey post hoc tests.

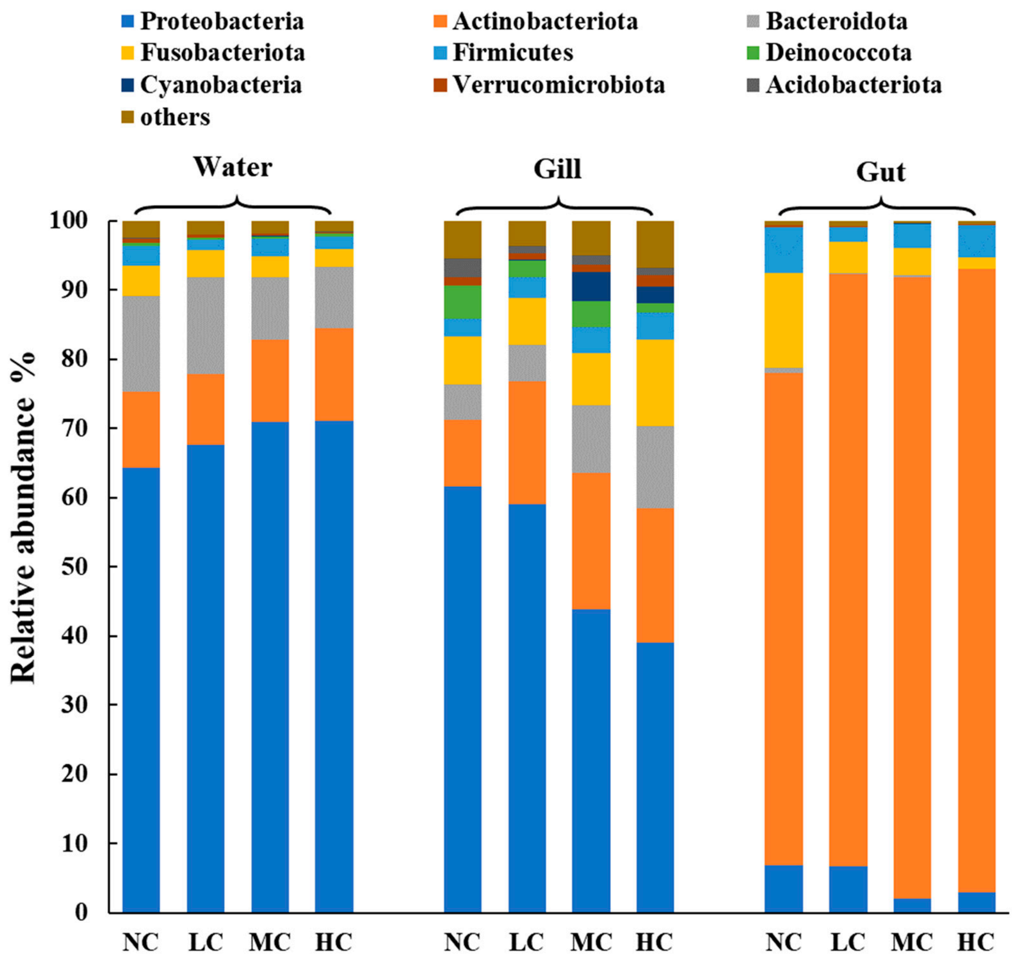

Figure S3. The relative abundance of the dominant phyla (relative abundance > 0.5%) of bacterial communities inhabiting different microhabitats in treatments with different *Chlorella pyrenoidosa* additions. NC, no addition; LC, low concentration; MC, medium concentration; HC, high concentration.

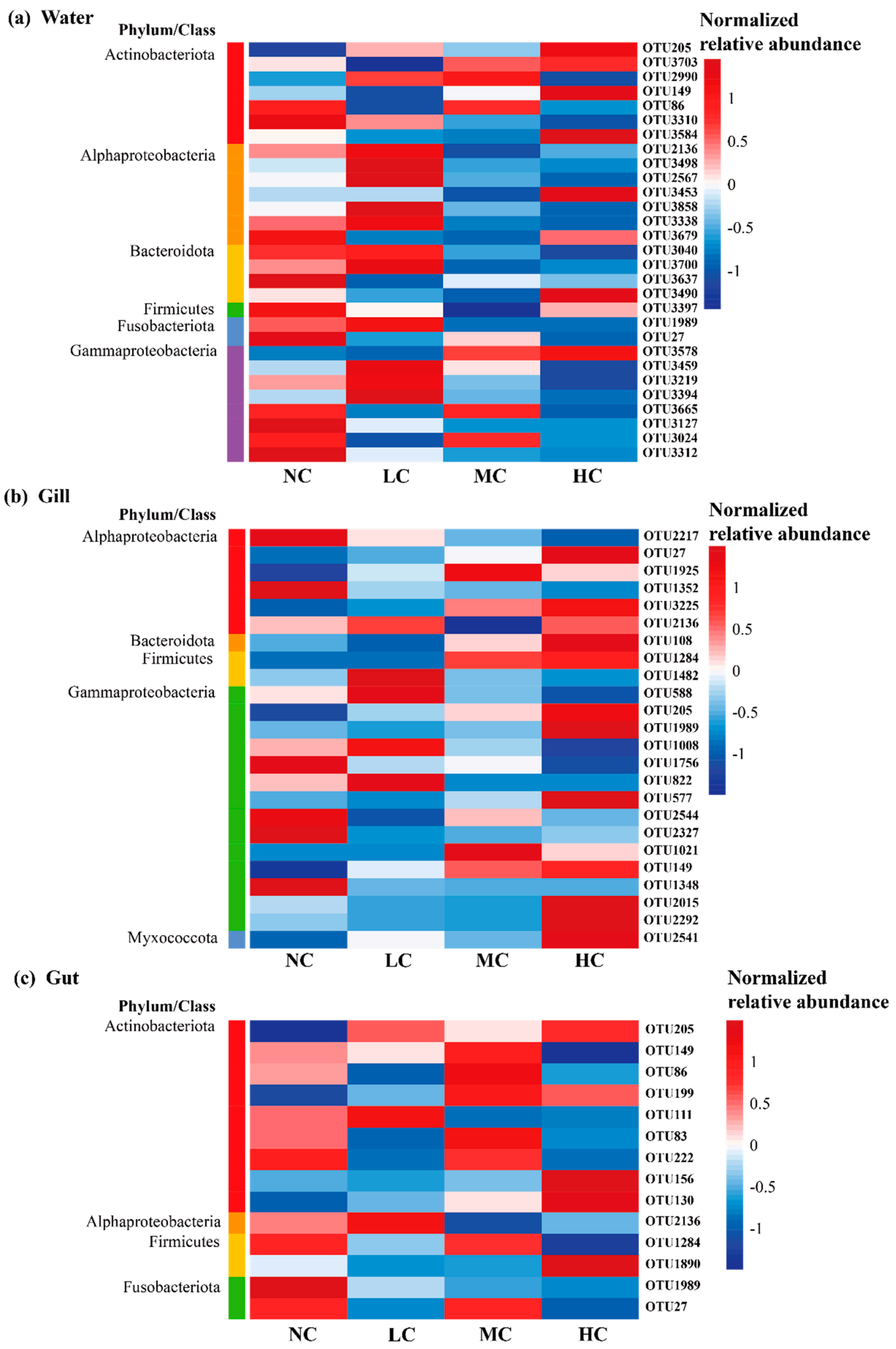

Figure S4. Heatmaps showing the relative abundance of dominant OTUs (mean relative abundance > 0.5%) across different treatments (different *Chlorella pyrenoidosa* additions) in water (a), gill (b) and gut (c).

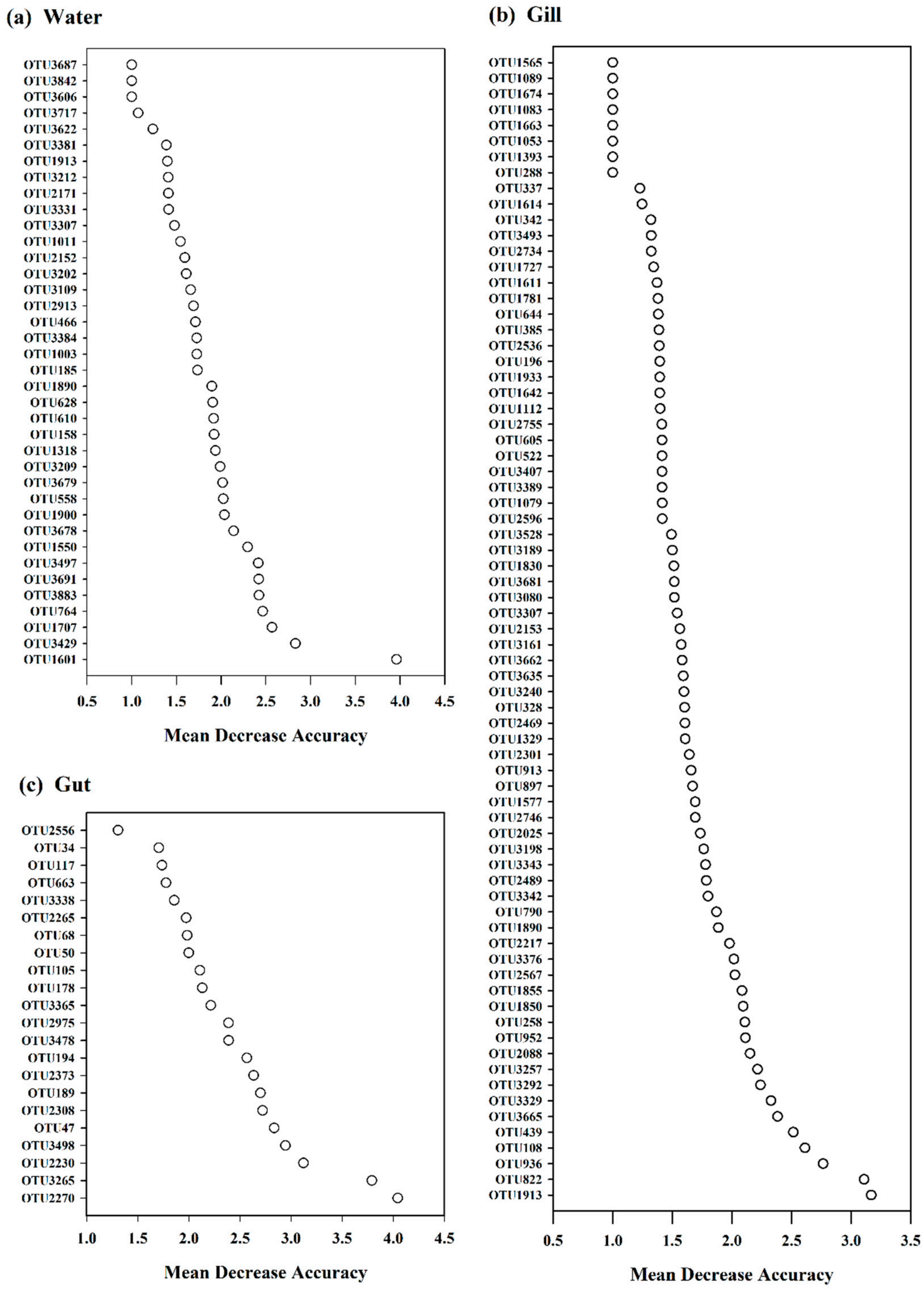

Figure S5. Bacterial taxa that are significantly influenced by the *Chlorella pyrenoidosa* addition in water, gill and gut detected by random forest models.
